# ﻿*Cryptanthawhippleae* (Boraginaceae), a new serpentine-adapted species endemic to northern California, U.S.A.

**DOI:** 10.3897/phytokeys.247.132060

**Published:** 2024-10-15

**Authors:** Michael G. Simpson, Dana A. York

**Affiliations:** 1 Department of Biology, San Diego State University, San Diego, California 92182, USA San Diego State University San Diego United States of America; 2 Botany Research Associate, California Academy of Sciences (CAS), 55 Music Concourse Drive, Golden Gate Park, San Francisco, California 94118, USA California Academy of Sciences San Francisco United States of America

**Keywords:** Boraginaceae, California, conservation, *Cryptanthawhippleae*, endemic, Klamath Mountains, serpentine, taxonomy

## Abstract

*Cryptanthawhippleae* D.A.York & M.G.Simpson (Boraginaceae) is described as new. This species is currently known to occur in serpentine barrens in the Shasta-Trinity National Forest of Siskiyou County, California, with one outlier population in possible serpentine of Lake County, California. The new species is most similar to *Cryptanthagrandiflora* and to *C.milobakeri*, these three likely each others’ closest relatives. All three have a relatively large corolla limb width and similar smooth, lance-ovate to ovate, marginally rounded, acuminate and abaxially transversely flattened nutlets. *Cryptanthawhippleae* differs from *C.grandiflora* in having a short, as opposed to a tall, stem height; bifurcate as opposed to trifurcate primary axis cymules; and typically 2–3 nutlets per fruit, as opposed to usually one nutlet per fruit. *Cryptanthawhippleae* differs from *C.milobakeri* also in having a short, versus tall, stem height; appressed-strigose and spreading-hispid stem vestiture, as opposed to strigose only or strigose and hirsute; calyx trichomes with two distinct vestiture types, these marginally appressed hirsute and medially hispid, as opposed to calyx trichomes of one type, dense, appressed to ascending, whitish sericeous; and 2–3 nutlets per fruit, as opposed to one nutlet per fruit. *Cryptanthawhippleae* is relatively rare and joins seven other *Cryptantha* species that are found on serpentine, either obligately or facultatively. Current molecular phylogenetic studies support the mostly convergent evolution of serpentine adaptation in *Cryptantha*, but additional studies are needed.

## ﻿Introduction

*Cryptantha* is a genus of annual or (only in some South America taxa) perennial herbs of the family Boraginaceae, subtribe Amsinckiinae [sensu Chacón et al. (2016)]. *Cryptantha* s.l. has been found to be non-monophyletic in several molecular phylogenetic studies (Hasenstab-Lehman and Simpson 2012; Weigend et al. 2013; Simpson et al. 2017a; Mabry and Simpson 2018). Based on these studies, the genus was recircumscribed and split from the genera *Eremocarya*, *Greeneocharis*, *Johnstonella* and *Oreocarya* by Hasenstab-Lehman and Simpson (2012), these results being confirmed by Simpson et al. (2017a) and Mabry and Simpson (2018). This updated classification has been consistently used, for example, in the *Jepson eFlora* [Jepson Flora Project 2024] of California vascular plants and in the treatments in the Flora Argentina project (Moroni et al. 2021; Moroni and Simpson 2022, 2023a, b, c). *Cryptantha* is currently recognised with 109 species and 124 minimum-ranked taxa, 63 of those species occurring in North America and 47 species in South America, with one taxon [Cryptanthamaritima(Greene)Greenevar.pilosa I.M.Johnst.] found on both continents (see Simpson et al. (2017b); Amsinckiinae Working Group (2024)).

Serpentine soils, specifically in northern California, are formed from ultramafic (meta-igneous) rocks that developed millions of years ago deep in the ocean floor. The soils are extremely high in heavy metals (i.e. nickel, iron and magnesium) and low in calcium and potassium. Serpentine soils are inhospitable to plants that have not evolved to tolerate the harsh conditions. Plants growing on serpentine outcrops tend to be slow-growing and isolated geographically and reproductively, thus evolving into new species (Kauffmann et al. 2022). Sawyer (2006) documented 200 neoendemic serpentine plants in north-western California. Serpentine-adapted species have a high rate of endemism (Harrison et al. 2004).

Seven species of *Cryptantha* have previously been identified as occurring on a serpentine substrate (Safford and Miller 2020; Table 1): *Cryptanthadissita* I.M.Johnst., *C.excavata* Brandegee, *C.flaccida* (Douglas ex Lehm.) Greene, *C.hispidula* Greene ex Brand, C.intermedia(A.Gray)Greenevar.intermedia, *C.mariposae* I.M.Johnst. and *C.milobakeri* I.M.Johnst. Three of these species are listed in the *Inventory of Rare and Endangered Plants of California* as **1B.1**, **1B.2** or **1B.3** (CNPS Inventory 2024; see Table 1). Of these seven species, we believe that C.intermediavar.intermedia is likely not serpenticolous and is, in fact, listed by the authors as **WI/IN**=Weak Indicator/Indifferent. However, we add the taxon C.clevelandiiGreenevar.clevelandii to the list, as some San Luis Obispo County populations of that taxon occur on serpentine (personal observation by the first author).

**Table 1.** Comparison of morphological features and rankings of known serpenticolous species of *Cryptantha*, plus *C.grandiflora*, a presumed close relative of *C.whippleae*. Morphological data are from Simpson and Kelley (2020), Simpson et al. (2021) and personal observations. Substrate rankings in bold are from rankings in Safford and Miller (2020): **BE/SI** = Broad Endemic/Strong Indicator; **SE** = Strong Endemic; **SI** = Strong Indicator; **WI** = Weak Indicator. Rarity rankings are from the CNPS Inventory (2024): **1B.1**=Rare or Endangered, Seriously threatened in California; **1B.2** Rare or Endangered, Moderately threatened in California; **1B.3**=Rare or Endangered, Not very threatened in California. *= Suggested rankings by the authors of this paper.

Taxon	Substrate, Rankings	Plant Height	Stem Vestiture	Inflorescence Bracts	Cymule Number	Corolla Limb Width	Fruiting Calyx Length	Calyx Vestiture	Nutlet No./Fruit	Nutlet Scuplturing, Shape	Nutlet Length	Style Extension
C.clevelandiivar.clevelandii (in part)	Mostly not serpentine (except San Luis Obispo Co), WI*	10–50 cm	Strigose or strigose and spreading-hispid	Generally absent	1–2	1–5 mm	3–4.5 mm	Two trichome types: marginally appressed hirsute; medially hispid	1–4	Smooth, lance-ovate, abaxially convex, margin rounded	1.5–2 mm	2/3–9/10 nutlet length
*C.dissita*	Serpentine, **BE/SI, 1B.2**	8–25 cm	Spreading-hirsute only	Absent (peduncle naked below)	(2)3	(4)5–8 mm	3.5–5.5(6) mm	Two trichome types: marginally appressed hirsute; medially hispid	(1)2–4	Smooth, lanceolate to lance-ovate, abaxially convex, margin rounded	1.8–2.2 mm	≥ nutlet length
*C.excavata*	Rarely serpentine, **WI**, **1B.1**	5–30 cm	Strigose or strigose and hirsute to hispid	Absent	2–3	3–5(6) mm	2–2.5 mm	Two trichome types: marginally appressed hirsute, medially sparsely hispid	1(2,3)	Tuberculate + papillate, lance-ovate, abaxially convex, margin rounded; areole cavity-like	2–2.4 mm	2/3–3/4 nutlet length
*C.flaccida*	Rarely serpentine, **WI**	15–50 cm	Strigose only	Absent	1–5	1–5(6) mm	3–4.5(5) mm	Two trichome types: marginally appressed hirsute, medially recurved or hook-tipped	1	Smooth, lance-ovate, abaxially convex (“plump”), margin rounded	1.8–2.3 mm	1/3–1/2 nutlet length
*C.grandiflora*	Not serpentine; rocky, clay, or volcanic soils	5–35 cm	Strigose and spreading-hispid	Rarely present, if so 1 at cymule base	3(1,2)	4–8 mm	3.5–5.5 mm	Two trichome types: marginally appressed hirsute; medially hispid	1(2)	Smooth, lance-ovate to narrowly ovate. abaxially flattened, margin rounded	1.8–2.8 mm	up to 3/4 nutlet length
*C.hispidula*	Serpentine, **SE**	10–50 cm	Strigose and spreading-hispid	Absent	2–3	3–6 mm	(2.5)3–4	Two trichome types: marginally appressed hirsute; medially hispid	1(2)	Smooth, lanceolate to lance-ovate, abaxially convex, margin rounded	1.7–2.2 mm	2/3 to 3/4 nutlet length
*C.mariposae*	Serpentine, **SE, 1B.3**	8–25 cm	Strigose and ascending to spreading-hispid	1–few, near base of cymules	1(2,3)	(2)3–6 mm	4–6(7) mm	Two trichome types: marginally appressed hirsute; medially hispid	(2)3–4	Tuberculate + papillate, lance-ovate to ovate, abaxially convex, margin rounded	1.9–2.2 mm	much > nutlet length
*C.milobakeri*	Serpentine, **SI**	10–50 cm	Strigose and spreading-hirsute to hispid	Absent or occasionally 1 near base of cymules	2–3	2–6 mm	3–5 mm	One trichome type: long, soft-tufted, appressed to ascending sericeous, often whitish	1(2)	Smooth, lance-ovate to ovate, abaxially flattened, margin rounded	1.5–2(–2.5) mm	2/3–3/4 nutlet length
*C.whippleae*	Serpentine, SE*, 1B*	3–8(15) cm	Strigose and spreading-hispid	Present at base of cymules	2(1)	3–6 mm	4–5.5 mm	Two trichome types: marginally appressed hirsute; medially hispid	2–3	Smooth, lance-ovate to ovate, abaxially flattened, margin rounded	1.6–2.6 mm	3/4 nutlet length

**Figure 1.**
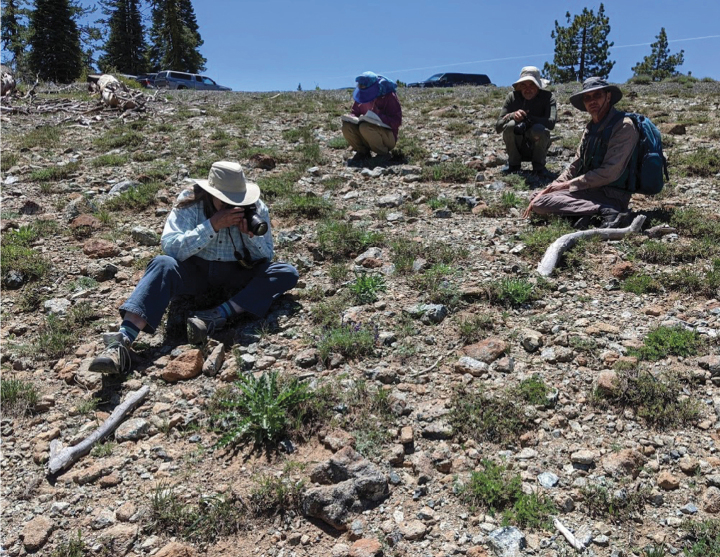
Site of holotype of *Cryptanthawhippleae*, a rocky, serpentine outcrop. Seen in this 18 June 2022 photograph, from left to right, are: Jennifer Whipple, Ellen Uhler, Michael Uhler and Dana York. Photo by Julie Kierstead.

**Figure 2.**
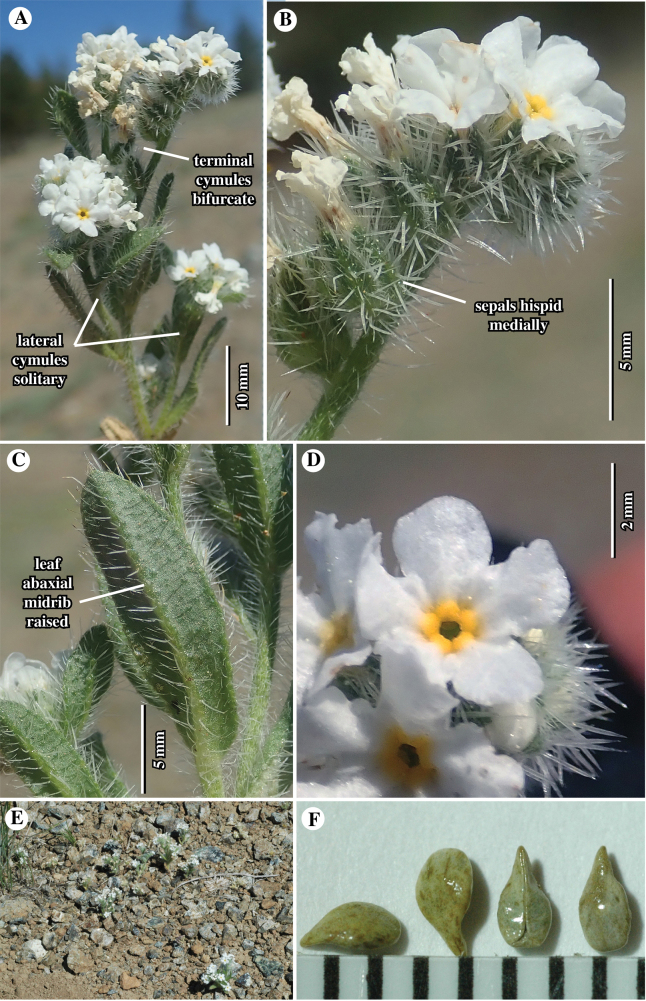
Field shots of *Cryptanthawhippleae* at holotype locality **A** upper part of plant. Note terminal bifurcate cymules at apex of primary stem; lateral cymules are solitary **B** close-up of a single cymule. Note hispid vestiture along sepal mid-ribs **C** ascendingly orientated stem leaf, abaxial surface showing hispid vestiture along raised mid-rib **D** close-up of corolla, showing yellow fornices and relatively large limb (this one ca. 5 mm wide) **E** several plants in the field at the type locality. Note small stature of plants and surrounding rocky, gravelly serpentine substrate **F** free nutlets (from various fruits), characteristically smooth and shiny, ovate to lance-ovate, abaxially transversely flattened, apically acuminate.

**Figure 3.**
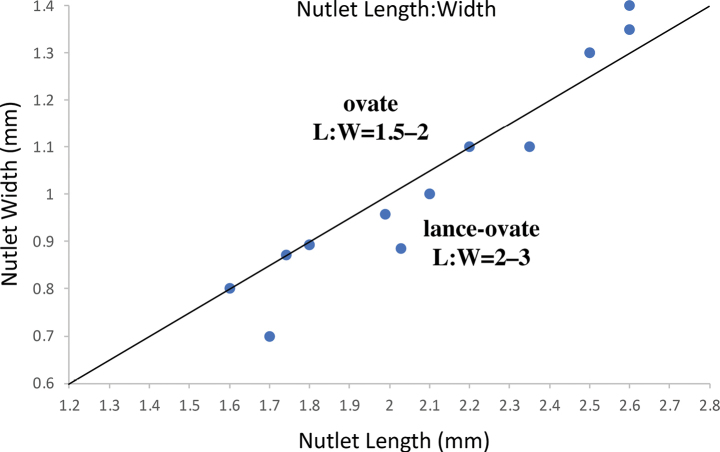
Graph of average length and width of nutlets of all known specimens of *Cryptanthawhippleae*, showing variation in size. Straight line (slope = 2) shows the demarcation between an ovate shape (length: width ratio 1.5–2) and a lance-ovate shape (length: width ratio 2–3). Note that nutlets of *C.whippleae* span between the two shape categories; terminology after Simpson (2019).

**Figure 4.**
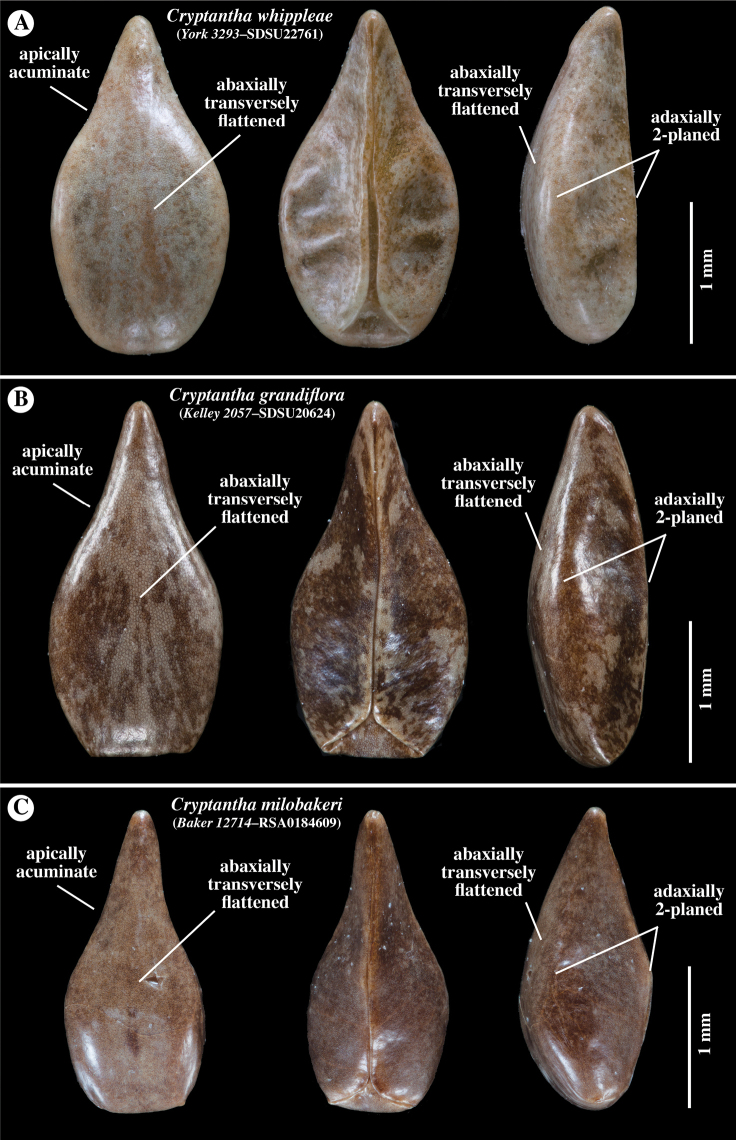
Comparison of representative nutlets of **A***Cryptanthawhippleae***B***Cryptanthagrandiflora* and **C***Cryptanthamilobakeri*. All are smooth and shiny, round-margined, apically acuminate, ranging from lance-ovate to ovate in shape, with a transversely flattened abaxial surface, a 2-planed adaxial surface, truncate to rounded base, rounded margins and contiguous ventral groove attachment scars, 2-forked at base delimiting a small to absent areole. Collector and accession numbers of specimens indicated.

**Figure 5.**
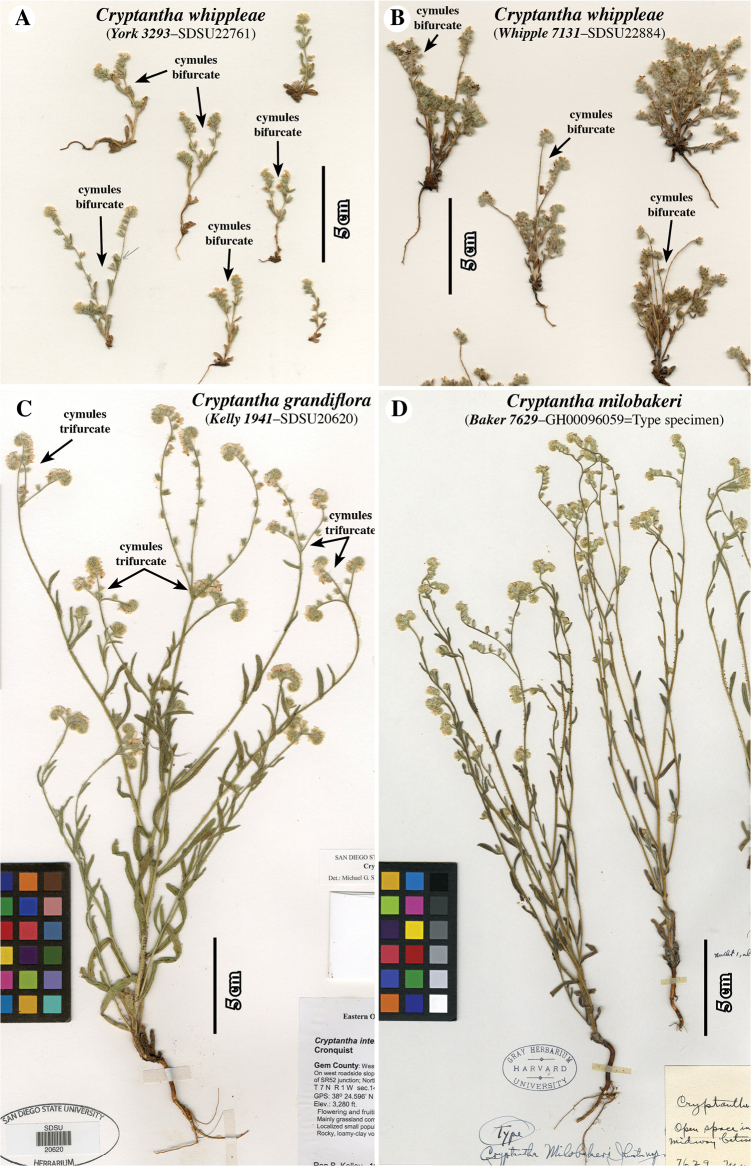
Herbarium specimen images of **A, B***Cryptanthawhippleae***C***Cryptanthagrandiflora* and **D***Cryptanthamilobakeri*, all imaged at the same scale. Note relatively small stature of *C.whippleae*, which typically has bifurcate terminal cymules (**A**) as opposed to trifurcate terminal cymules in *C.grandiflora* (**C**); cymules of *C.milobakeri* (**D**) can be bifurcate or trifurcate. Collector and accession numbers of specimens indicated.

**Figure 6.**
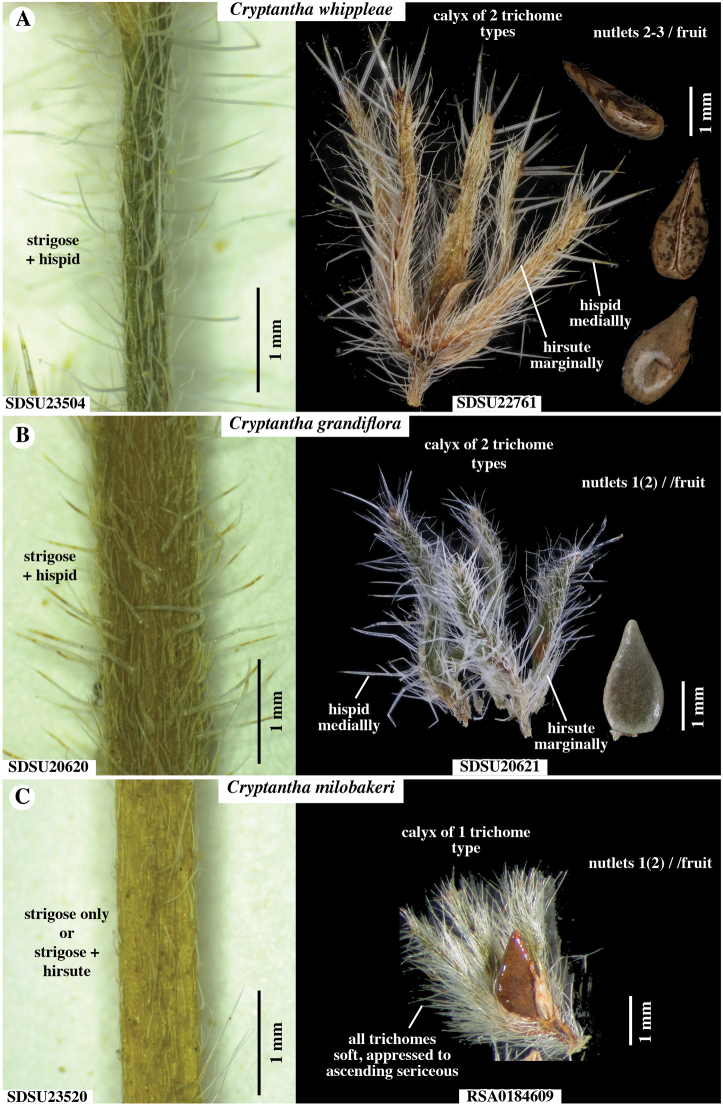
Comparison of stem vestiture (left) and fruits (right) of **A***Cryptanthawhippleae***B***Cryptanthagrandiflora* and **C***Cryptanthamilobakeri*. Stems of *C.grandiflora* and *C.whippleae* are similarly both strigose and spreading hispid. Stems of *C.milobakeri* are mostly strigose, sometimes also with spreading, fine-hirsute trichomes. Fruiting calyces of *C.grandiflora* and *C.whippleae* are marginally appressed hirsute and coarse hispid along the mid-rib. Those of *C.milobakeri* characteristically have one type of trichomes, consisting of appressed to ascending, soft, whitish, hirsute trichomes. Accession numbers of specimens indicated.

A distinct form of *Cryptantha* was discovered in the Klamath Mountains, near Mt. Eddy of the Shasta-Trinity National Forest of California (see Figs 1–4) and several voucher specimens were collected. Additional specimens of this form were identified from previously deposited herbarium collections and these all were compared with morphologically similar taxa (Figs 4–6). From these studies, we found this form to be unique in the genus, warranting its recognition as a new species, based on a taxonomic (morphologic) concept (Cronquist 1978, 1988).

## ﻿Methods

**Figure 7.**
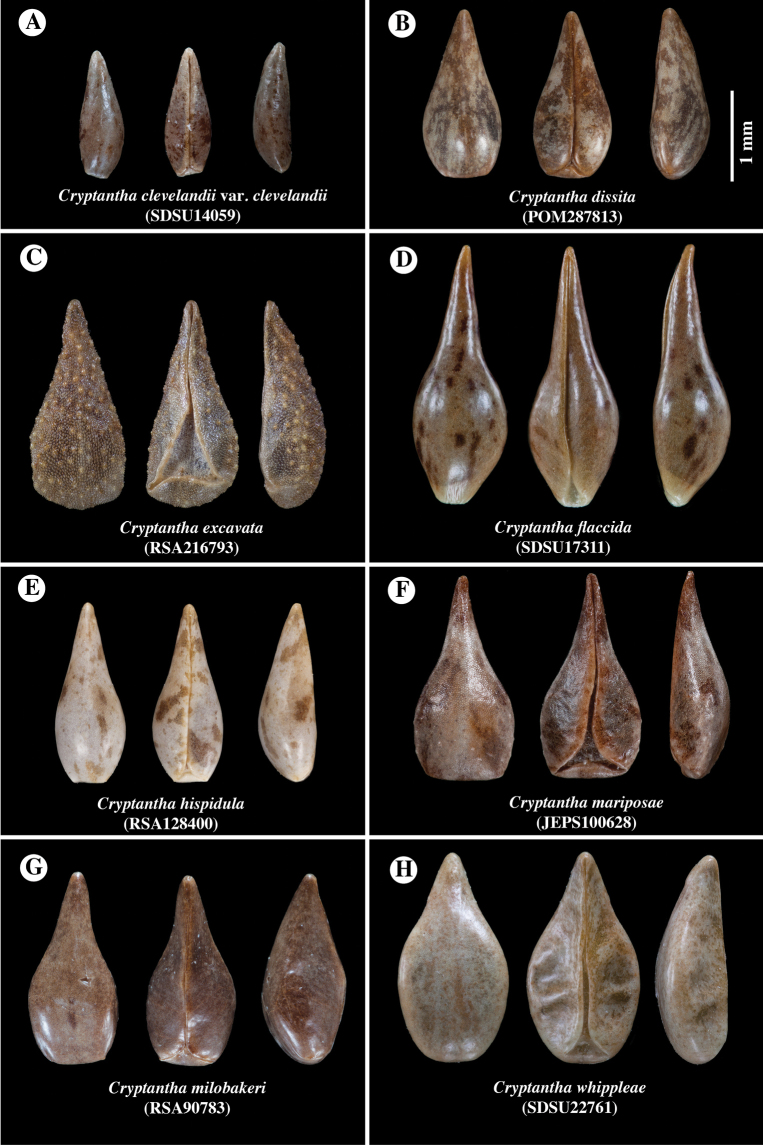
Comparison of nutlets of the eight *Cryptantha* species that occur on a serpentine substrate, in (left to right) dorsal, ventral and lateral views with herbarium accession numbers of samples listed. All nutlet images are shown to scale **A**C.clevelandiivar.clevelandii**B***C.dissita***C***C.excavata***D***C.flaccida***E***C.hispidula***F***C.mariposae***G***C.milobakeri***H***C.whippleae*. Accession numbers of specimens indicated.

**Figure 8.**
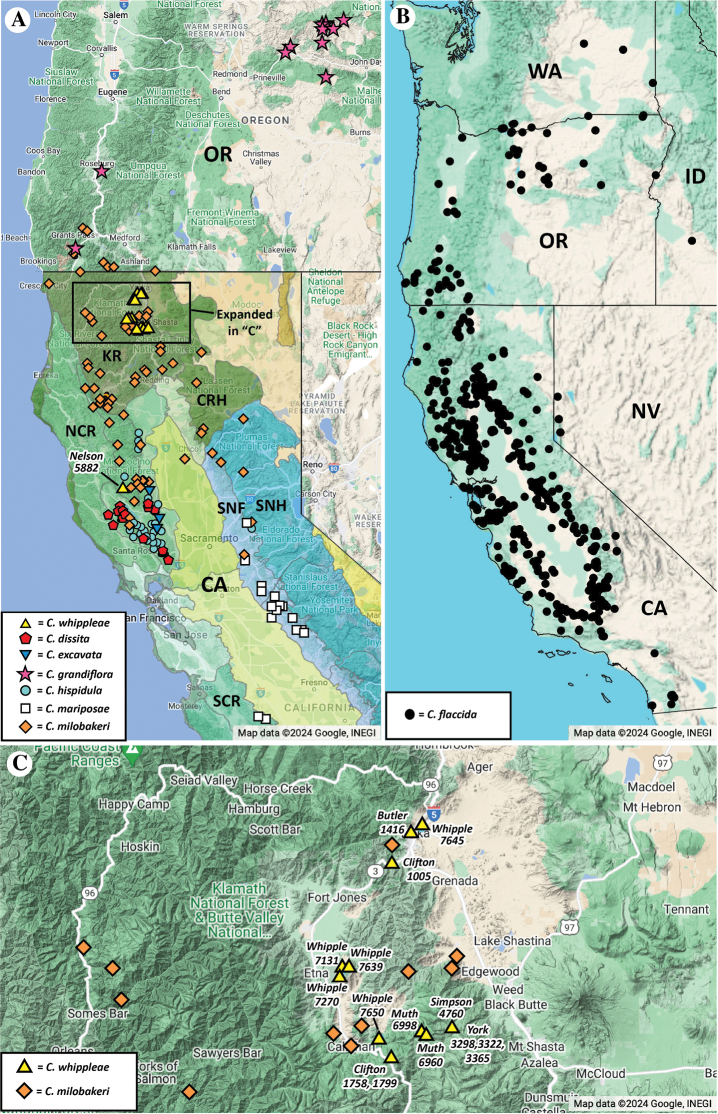
Distribution maps **A** map showing the overall distribution of *C.whippleae*, of presumed close relatives *C.grandiflora* (red stars) and *C.milobakeri* (orange diamonds) and of other serpentine *Cryptantha* species in California (CA) and Oregon (OR) (minus *C.clevelandii*var.c., which occurs further south); see legend for symbols. Note disjunct locality of *C.whippleae* in Lake County, California (*Nelson 5882*). California bioregions after Jepson Flora Project (eds.) (2024): CRH = Cascade Range Highlands; KR = Klamath Ranges; NCR = North Coast Ranges; SCR = South Coast Ranges; SNF = Sierra Nevada Foothills; SNH = Sierra Nevada Highlands **B** distribution map of *Cryptanthaflaccida* (black dots), another serpentine taxon, albeit a weak indicator, sensu Safford and Miller (2020). This species is plotted separately because of its relatively widespread distribution in California (CA) and scattered in Idaho (ID), Oregon (OR), Washington (WA) and western Nevada (NV) **C** close-up of rectangle in “**A**”, showing known sites of herbarium vouchers of *Cryptanthawhippleae* (yellow triangles) in the Mt. Eddy region of Siskiyou County, California, labelled by collector/collection number (type at *York 3365*). Herbarium voucher sites of *Cryptanthamilobakeri* (orange diamonds) in the same region are also shown. All maps from Google 2024, INEGI Data.

We collected additional specimens of the presumed new species in the Mt. Eddy region of Shasta-Trinity National Forest. An earlier collection was designated to be the type (holotype and isotypes) of the new species. In addition, we identified *Cryptantha* specimens that fit this new taxon from herbarium vouchers (listed as paratypes) from Cal Poly Humboldt (**HSC**), Pacific Union College Herbarium (**PUA**), California Botanic Garden (**RSA**), San Diego State University (**SDSU**) and University of California, Berkeley (**UC**); acronyms after Thiers (2024). These specimens were studied morphologically using a dissecting microscope and a spreadsheet of morphological characters was made to generate a description of the new taxon. Fruits were examined from all specimens. Fruiting calyx length, nutlet number per fruit and nutlet size (length, depth and maximum width) were quantified. Mean nutlet length versus maximum width for all known specimens was graphed (Fig. 3) and evaluated in terms of qualitatively evaluating nutlet shape as ovate (with a length: width ratio = 1.5–2) versus lance-ovate (length: width ratio = 2–3), terminology after Simpson (2019). In order to illustrate their similarities and differences, fruiting calyces, nutlets and stems of the new species and of specimens of *Cryptanthagrandiflora* Rydberg [C.intermedia(A. Gray)Greenevar.grandiflora (Rydberg) Cronquist], *C.milobakeri* and six additional serpenticolous *Cryptantha* taxa were imaged using a Macropod Pro 3D camera system (Macroscopic Solutions, East Hartford, CT, USA) or an Infinity 2 camera on an Olympus SZ61 boom-mounted dissecting microscope (Figs 4, 6, 7). Representative herbarium specimens were also imaged for comparative purposes (Fig. 5). From collection label data of *C.whippleae* and from georeferenced specimen data available on the CCH2 (2024), we prepared distribution maps (Fig. 8A–C) for this new taxon and for *Cryptantha* species that occur on serpentine (minus *C.clevelandii*) and for the morphologically similar *C.grandiflora*. Maps (Fig. 8A, C) were prepared using the Berkeley Mapper tool (https://ucjeps.berkeley.edu/consortium/load_mapper_multi.html) or (Fig. 8B) using R v.4.3.1, occurrence points plotted on a custom Google Map (Kahle and Wickham 2013).

## ﻿Results

Based on our studies of specimens of this new taxon and of morphologically similar *Cryptantha* species, we describe here a new species.

### ﻿Taxonomic treatment

#### ﻿ Cryptantha whippleae

Taxon classificationPlantaeBoraginalesBoraginaceae

D.A.York & M.G.Simpson sp. nov.

458B44CE-2D0C-5734-B363-EBEBEDBE3B34

urn:lsid:ipni.org:names:77350290-1

##### Note.

Specimens cited indicate herbarium accession numbers, acronyms after Thiers (2024).

##### Type.

United States • California, Siskiyou County, Shasta-Trinity National Forest, The Eddys, ridge between China Mtn. and Mount Eddy, a few metres E of the county line, ca. 210 m N-NW of Parks Creek Trailhead. Annual with white (appendages yellow) flowers, rare, growing in serpentine soils on a S-facing, exposed, gravelly slope, serpentine soil, gravelly, associated taxa: *Danthoniaunispicata*, *Eriogonumsiskiyouense*, Eriogonumumbellatumvar.humistratum, Eriogonumumbellatumvar.nelsoniorum, *Eriophyllumlanatum*, *Festucaidahoensis*, *Penstemonroezlii* and *Pinusjeffreyi*. 41.34458, -122.53863, 2100 m (6888 feet) elevation. 18 June 2022, *D. York 3365 with Julie Kierstead*, *Ellen Uhler*, *Michael Uhler and Jennifer Whipple* (***holotype*: CAS**1352444; ***isotype*: HSC**105848).

##### Diagnosis.

*Cryptanthawhippleae* is similar to *C.grandiflora* in having a relatively wide corolla limb [3–6 mm wide in *C.whippleae*; 4–8 mm wide in *C.grandiflora*] and in the size, shape and sculpturing of nutlets, differing in having a mostly shorter plant height [3–8(15) cm tall in *C.whippleae* versus 5–35 cm tall in *C.grandiflora*], in cymule branching [bifurcate or rarely solitary in *C.whippleae* versus trifurcate in *C.grandiflora*] and in having more nutlets per fruit [2–3 in *C.whippleae* versus (2) in *C.grandiflora*]. *Cryptanthawhippleae* is similar to *C.milobakeri* in corolla limb width [3–6 mm wide in *C.whippleae*; 2–6 mm wide in *C.milobakeri*] and in the size, shape and sculpturing of nutlets, differing in having a shorter height [3–8(15) cm tall in *C.whippleae* versus 10–50 cm tall in *C.milobakeri*], in calyx vestiture [with two distinct trichome types in *C.whippleae* versus a single trichome type in *C.milobakeri*] and in having more nutlets per fruit [2–3 in *C.whippleae* versus 1(2) in *C.milobakeri*].

##### Description.

(Figs 1–6). ***Plants*** annual, 3–8(15) cm tall, grey-green. ***Root*** a single taproot, not reddish. ***Stems*** erect, vegetative primary stem usually terminating in an inflorescence of bifurcate cymules (rarely of a solitary cymule), 0–2 lateral branches arising from base and/or middle region of primary stem, these usually terminating in a solitary cymule, stem surface both strigose, with trichomes antrorsely appressed, abruptly up-turned at base, ca. 0.5 mm long, and hispid, these trichomes spreading to inclined, ca. 1–1.5 mm long, ca. 0.05 mm wide proximally, mostly swollen at base, surfaces minutely scaberulous, all trichomes white, sharply tapered. ***Leaves*** alternate, those at plant base 4–7 in number, densely clustered, horizontal to ascending in upper cauline leaves, often brownish at anthesis, 4–10 mm × 1.5–3 mm at widest point, oblanceolate to obovate, those along aerial primary stem 0–4 in number, regularly spaced, ascending to appressed, green-grey, 7–15 mm × 1.5–3 mm at widest point, sessile, oblanceolate, oblance-ovate or narrowly oblong, base cuneate, apex obtuse to rounded, typically subtending lateral branches and base of cymule units, those above base often showing apparent evidence of herbivory; adaxial surface with mid-rib sunken, margins hispid, trichomes white, 1–2 mm long, ascending to appressed, trichome bases bulbous and prominently pustulate, pustules of 2 concentric rows of white to transparent, radially oblong cells; abaxial surface with strongly ridged mid-rib, hispid especially along mid-rib, trichomes similar to those of adaxial surface, but less prominently pustulate. ***Inflorescence*** with bifurcate (paired) cymules arising from the primary stem (cymules rarely solitary), a flower/fruit typically found at the junction of the cymules, with 1–2 additional solitary cymules branching from lower primary stem, cymules 20–65 mm long including basal axis, lowest flowers not touching at maturity, inflorescence bracts leaf-like, typically present at and slightly above cymule bases. ***Flowers*** with pedicels ca. 0.5 mm, hirsute and hispid, trichomes 0.5–1 mm, horizontal to ascending, subtending leaf-like flower bracts subtending only lowest 1–2 flowers, upper flowers lacking bracts. ***Calyx*** at anthesis 1.5–2 mm, in fruit 4–5.5 mm, ovoid, slightly constricted above middle, sepals distinct, lanceolate, erect, apices erect to recurved, abaxial mid-rib thickened, surface along sepal sides with trichomes straight, soft hirsute, inclined to ascending, 0.5–1.5 mm long, the raised mid-rib and sepal apex hispid, with trichomes horizontal to inclined, 1–1.5 mm long, ca. 0.5 mm wide near base, bulbous-based and often pustulate, trichome surface scaberulous, adaxial sepal surface glabrous basally, with appressed short trichomes apically. ***Corolla*** showy, white with yellow fornices, rotate, tube as long as calyx, limb 3–6 mm wide. ***Androecium*** of five stamens, attached at the same level ca. 2/3 along corolla tube between and below fornices; anthers ca. 0.5 mm long, ellipsoid, dithecal, introrsely dehiscent, dorsifixed; filaments filiform, ca. 0.1 mm long. ***Gynoecium*** four-lobed, lobes ca. 0.4 mm long, widely ellipsoid to oblong, style gynobasic, ca. 0.8 mm long. ***Nutlets*** 2–3 per fruit, 1.6–2.6 mm × 0.8–1.4 mm wide at widest point, length: width ratio 1.6–2.6, homomorphic, lance-ovate to ovate, margins rounded, base broadly rounded to truncate, apex short-acuminate, extreme tip acute-rounded, abaxial surface transversely flattened, slightly curved longitudinally, spinal ridge absent, adaxial surface 2-planed convex, both surfaces smooth and shiny, brown to grey-brown, often dark brown mottled, attachment scar ventral groove in lateral view relatively straight, in face view, edges slightly raised, abutted apically, 2-forked at base, contiguous or delimiting small areole. ***Gynobase*** at maturity ca. 1/2 height of nutlets, style extending to ca. 3/4 height of mature nutlets. Abortive nutlets 1–2, tan to brown, lanceoloid to ellipsoid, position relative to inflorescence axis variable.

##### Distribution and habitat.

*Cryptanthawhippleae* is endemic to northern California, USA, ranging in elevation from ca. 800 to 2200 m. It occurs in open, rocky, serpentine substrate habitats (Figs 1, 2E). All but one of the known specimens occur in Siskiyou County. The sole Lake County specimen (*Nelson 5882*) is possibly on serpentine, but substrate type was not recorded on the label (see Discussion).

##### Phenology.

Based on herbarium specimen records, *Cryptanthawhippleae* flowers from late May to early August. Fruits typically mature within a few weeks after flowering.

##### Rarity and conservation status.

*Cryptanthawhippleae* is currently known from 15 collections in only 12 specific localities, all in northern California. Pending further surveys, we recommend that it be ranked as **1B** (“rare, threatened or endangered in California and elsewhere”) using the California Native Plant Society Inventory Rankings (CNPS Inventory 2024).

##### Etymology.

The epithet is named after Jennifer J. *Whipple*, an avid collector in the Mount Eddy/Scott Valley region and a retired Yellowstone National Park botanist. The epithet *whippleae* can be pronounced whíp-pul-ee as a commemorative, using the female genitive ending -*ae* and following Anglicised Latin (Stearn 1993).

##### Suggested common name.

We suggest Whipple’s Cryptantha as a common name.

##### Paratypes

**(arranged alphabetically by county, then by collector/collection number).** United States, California • Lake County: along Forest Service Rd. 17N16, 3.1 mi. E of Bear Creek Ranger Station, Chaparral, 39.326214, -122.786329, 1220 m elevation, 24 June 1980, *T. W. Nelson & Jane Nelson 5882* (**HSC**202692!) • Siskiyou County: Dry hill near Yreka, 41.73234, -122.64111 [estimated from label locality data], 804 m elevation [estimated from label locality data], 27 May 1910, *G. D. Butler 1416* (**RSA**0153874!, **UC**163852!) • Local landmark: Hayden Cabin. China Mt Quad, Mountain or Hillside Slopes, Slope Position: Upper Third, Vertical Slope Shape: Convex, Horizontal Slope Shape: Convex, Very Gravelly texture composed mainly of serpentine with a colluvial origin, 41.285611, -122.694556, 1737 m elevation, 2 July 1978, *Clifton & Ground 1758* (**PUA**-CardNumber15387!) • Local landmark: Hayden Cabin. China Mt Quad, Mountain or Hillside Slopes, Slope Position: Upper Third, Vertical Slope Shape: Convex, Horizontal Slope Shape: Convex, Gravelly texture composed mainly of serpentine with a colluvial origin, 41.285611, -122.694556, 1737 m elevation, 2 July 1978, *Clifton & Ground 1799* (**PUA**-CardNumber15438!) • Near Rock Fence Lake. China Mt Quad, close to the town of Callahan, Slope Position: Middle Third, Vertical Slope Shape: Smooth, Horizontal Slope Shape: Smooth, Gravelly texture composed mainly of serpentine with a colluvial origin, 41.336528, -122.609111, 2100 m elevation, 1 August 1978, *G. J. Muth 6998* (**PUA**-CardNumber14174!) • Local landmark: Cory Peak. China Mt Quad, Mountain or Hillside Slopes, Slope Position: Upper Third, Vertical Slope Shape: Smooth, Horizontal Slope Shape: Smooth, Very Gravelly texture composed mainly of serpentine with a colluvial origin, 41.333139, -122.603861, 2196 m elevation, 1 August 1978, *G. J. Muth 6960* (**PUA**-CardNumber14173!) • The Eddy’s, ca. 30 metres northwest of Pacific Crest Trail, near Parks Creek Trailhead, along old, compacted road, Open, rocky alpine vegetation, tan, clay loam of rocky, gravelly, serpintine outcrop, annual herb, 6 cm tall, corolla white with yellow centre (fornices), limb 4–5 mm broad, Not common. Ca. 40 individuals seen a few yards (metres) north on east side of road. Leaf material preserved in silica gel for genetic studies, 41.34464, -122.53864, 2099 m elevation, 28 June 2021, *M. G. Simpson & Lee M. Simpson 4760* (**SDSU**23504!) • Scott Valley, Weston Gulch, barren serpentine ridge, 41.462668, -122.825083, 990 m elevation, 14 June 2015, *J. J. Whipple 7131* (**SDSU**22884!) • Scott Valley, below Denny Point on hillside, Open serpentine north facing slope with scattered junipers, 41.4599, -122.828517, 990 m elevation, 31 May 2016, *J. J. Whipple 7270* (**SDSU**23523!) • Slopes above Scott Valley below Denny Point, barren rabbitbrush steppe on serpentine, 41.462683, -122.825, 990 m elevation, 1 June 2019, *J. J. Whipple 7639* (**SDSU**23524!) • China Hill by Yreka, serpentine barren, 41.743683, -122.614983, 900 m elevation, 3 June 2019, *J. J. Whipple 7645* (**SDSU**23525!) • Klamath National Forest, slope of Schneider Hill off of Masterson Road, 1.6 miles (2600 m) from Gazelle Callahan Road, Open serpentine barren, 41.32675, -122.726233, 1095 m elevation, 15 June 2019, *J. J. Whipple 7650* (**SDSU**23527!) • Shasta-Trinity National Forest, The Eddys, ridge between China Mtn. and Mount Eddy a few metres E of the county line, ca. 210 m N-NW of Parks Creek Trailhead, growing in serpentine soils on a S-facing, exposed, gravelly slope, serpentine soil, gravelly, A rare annual with white (appendages yellow) flowers, 41.34458, -122.53863, 2100 m elevation, 5 July 2016, *D. York 3293* (**SDSU**22761!) • Shasta-Trinity National Forest, The Eddys, ridge between China Mtn. and Mount Eddy a few metres E of the county line, ca. 210 m N-NW of Parks Creek Trailhead. A rare annual with white (appendages yellow) flowers, growing in serpentine soils on a S-facing, exposed, gravelly slope. 41.34461, -122.53862, 2100 m elevation, 10 July 2017, *D. York 3322* (**CAS**1352445!, **HSC**105849!).

### ﻿Key to the eight serpenticolous *Cryptantha* species

Key to the eight serpenticolous *Cryptantha* species, including *C.whippleae*, plus the non-serpenticolous, but presumed close relative *C.grandiflora*. Key modified from the *Jepson eFlora* (Simpson et al. 2021), only pertinent couplets included. (See Figs 4, 7 for comparison of nutlet morphology.)

**Table:** 

1	Nutlet(s) all smooth	**2**
–	Nutlets rough, variously papillate and tuberculate	**8**
2	Calyx trichomes both straight and hooked-tipped; nutlets 1	***C.flaccida***
–	Calyx trichomes straight to curved, not hook-tipped; nutlets 1–4	**3**
3	Calyx abaxially with ± single trichome type, generally long, soft, appressed to ascending, whitish sericeous, mid-vein trichomes slightly longer, but not hispid; nutlets 1(2)	***C.milobakeri***
–	Calyx abaxially with 2 trichome types, marginally appressed hirsute, medially spreading, ascending or reflexed hispid; nutlets 1–4	**4**
4	Nutlets lance-ovate to ovate, abaxially transversely flattened	**5**
–	Nutlets lance-ovate to lanceolate, abaxially convex	**6**
5	Plants 5–35 cm tall; terminal cymules trifurcate; nutlets 1(2)	***C.grandiflora***
–	Plants 3–8 cm tall; terminal cymules difurcate to rarely solitary; nutlets 2–3	***C.whippleae***
6	Stems unbranched or few-branched near base; leaves crowded proximally, subequal above; distal peduncle axis without bracts; nutlets (1)2–4	***C.dissita***
–	Stems branched throughout; leaves reduced distally, not congested proximally; distal peduncle axis typically with bracts; nutlets 1–2	**7**
7	Calyx in fruit appressed to axis; nutlet adaxially ± flattened, abaxially convex, not round in cross-section basally; generally non-serpentine, sedimentary based substrate (serpentine in San Luis Obispo County)	**C.clevelandiivar.clevelandii**
–	Calyx in fruit not appressed to axis; nutlet adaxially and abaxially ± rounded to convex, basally ± round in cross-section; obligate serpentine endemic	***C.hispidula***
8	Nutlets 1(2–3), densely papillate and tuberculate; apex narrowly acute; attachment scar areole deeply triangular in proximal half, cavity-like	***C.excavata***
–	Nutlets (2)3–4, densely papillate, sparsely tuberculate; apex long-acuminate; attachment scar areole relatively shallow, small at base, not cavity-like	***C.mariposae***

## ﻿Discussion

Where substrate data were recorded, all known specimens of *Cryptanthawhippleae* are reported in open, rocky, serpentine, corresponding to a strict endemic (“**SE**”) in the classification of Safford and Miller (2020). All but one of the collections of *Cryptanthawhippleae* cited here are centred at or near Mt. Eddy in Siskiyou County, California, including the holotype/isotypes *York 3365* (see Fig. 8A, C). However, one collection was discovered that is disjunct in range: *Nelson 5882*, of Lake County, California (see map, Fig. 8A). We confidently identified this specimen as *Cryptanthawhippleae*, as it fits all the morphological parameters of the species. The label information of the *Nelson 5882* specimen lists the sample as occurring on “chaparral,” with no reference to the substrate type, although it possibly came from serpentine since Nelson collected plants nearby on the same day citing serpentine on the labels (CCH2 2024). Cardace et al. (2013) (fig. 1, p. 48) map out several serpentine outcrops in Lake County, California.

*Cryptanthawhippleae* now adds an eighth, definitive serpenticolous species in the genus (Table 1). Of these eight taxa, phylogenetic relationships are known to date for only two: *Cryptanthaflaccida* and *Cryptanthamariposae*, these belonging to two distantly related clades (Simpson et al. 2017a; Mabry and Simpson 2018). Relationships of the remaining serpentine-adapted *Cryptantha* are uncertain, but there is no indication from taxonomic studies (Johnston 1925, 1939) that any are each others’ closest relative, except for *C.whippleae* being a likely close relative to the serpenticolous *C.milobakeri* and of the non-serpenticolous *C.grandiflora* (see below). Other than *C.milobakeri* and *C.whippleae*, our working hypothesis is that the serpenticolous *Cryptantha* taxa evolved adaptations to that rock substrate type independently.

*Cryptanthawhippleae* joins 21 additional species (23 minimum-ranked taxa) of North American members of *Cryptantha* with obligately smooth-nutlets (Amsinckiinae Working Group 2024). The taxa of *Cryptantha* with smooth nutlets are generally more difficult to distinguish from one another than those with “rough” (tuberculate and/or papillate) nutlets given the absence of diagnostic sculpturing surface features. Of these, *C.grandiflora*, *C.milobakeri* and *C.whippleae* are most similar in nutlet morphology and all share a relatively wide corolla limb. These three species all have smooth and shiny, lance-ovate to ovate, short-acuminate, abaxially transversely flattened (gently curving longitudinally) and adaxially 2-planed convex nutlets, a spinal ridge absent, the attachment scar a narrow ventral groove, closed or delimiting a small, basal areole, that of *C.whippleae* often slightly larger (Fig. 4). We believe that these species likely belong to the same phylogenetic clade, but they have yet to be sequenced. [It should be noted that *Cryptanthatorreyana* has nutlets similar to the above three (although relatively wider), but has a consistently small corolla limb width (1–2 mm); its closest relative is the “rough” nutlet species *C.ambigua* (Simpson et al. 2017a; Mabry and Simpson 2018)] *Cryptanthawhippleae* shows some variation in nutlet size and shape. Nutlets range from 1.6 to 2.6 mm long and 0.7 to 1.4 mm wide (at widest point). The shape ranges from lance-ovate to ovate, generally being near the boundary between these two (arbitrary) morphological terms (Fig. 3). As summarised in the Diagnosis, *Cryptanthawhippleae* differs from *C.milobakeri* in plant height, stem and calyx vestiture and nutlet number. It differs from *C.grandiflora* in plant height, inflorescence cymule number and nutlet number (see Figs 4–6 and Table 1 for comparisons).

We note anecdotally the observation of apparent herbivory of the basal leaves of some *Cryptanthawhippleae* specimens. Herbivory of *Cryptantha* species has been documented in South America (Villagrán et al. 2003; Echeverría et al. 2020), but requires further documentation in the North American continent.

In conclusion, we list *Cryptanthawhippleae* as a serpentine endemic (Table 1), given that all are cited on collection labels to occur on that substrate, except for the *Nelson 5882* specimen, which we think could be. It is possible, with the publication of this paper, that other specimens of *Cryptanthawhippleae* will show up in herbaria or from subsequent collections between the Shasta-Trinity region and the Lake County population. Surveys at and around the disjunct *Nelson 5882* specimen of Lake County will be valuable in order to confirm the occurrence of *C.whippleae* on a serpentine substrate. We also hope to obtain data on the interrelationships of populations from future molecular phylogenetic studies. The discovery of this new species highlights the need for additional taxonomic work on the flora of California, both from field collections and study of existing herbarium specimens.

## Supplementary Material

XML Treatment for Cryptantha whippleae

## ﻿Additional information

### Author ORCIDs

Michael G. Simpson https://orcid.org/0000-0002-6197-2132

Dana A. York https://orcid.org/0000-0002-5843-3306

### Data availability

All of the data that support the findings of this study are available in the main text.

## References

[B1] Amsinckiinae Working Group (2024) Systematics of Amsinckiinae (Boraginaceae): The Popcornflowers. https://plants.sdsu.edu/amsinckiinae

[B2] Cardace D Hoehler T McCollom T Schrenk M Carnevale D Kubo M Twing K (2013) Establishment of the Coast Range ophiolite microbial observatory (CROMO): Drilling objectives and preliminary outcomes. Scientific Drilling 16: 45–55. 10.5194/sd-16-45-2013

[B3] CCH2 (2024) Consortium of California Herbaria CCH2 Portal. https://cch2.org/portal/index.php [accessed 19.06.2024]

[B4] Chacón J Luebert F Hilger HH Ovcinnikova S Selvi F Cecchi L Guilliams CM Hasenstab-Lehman K Sutorý K Simpson MG Weigend M (2016) The borage family (Boraginaceae s.str.): A revised infrafamilial classification based on new phylogenetic evidence, with emphasis on the placement of some enigmatic genera. Taxon 65(3): 523–546. 10.12705/653.6

[B5] Cronquist A (1978) Once again, what is a species? In: Ramberger JA (Ed.) Biosystematics in Agriculture.Allanheld & Osmun, Montclair, NJ, 3–20.

[B6] Cronquist A (1988) The Evolution and Classification of Flowering Plants. Ed. 2. New York Botanic Garden, New York.

[B7] Echeverría J Paniagua-Zambrana NY Bussmann RW (2020) *Cryptanthahispida* (Phil.) Reiche Boraginaceae. In: Paniagua-Zambrana NY Bussmann RW (Eds) Ethnobotany of the Andes.Springer International Publishing, Cham, 1–2. 10.1007/978-3-319-77093-2

[B8] Harrison S Safford H Wakabayashi J (2004) Does the age of exposure of serpentine explain variation in endemic plant diversity in California? International Geology Review 46(3): 235–242. 10.2747/0020-6814.46.3.235

[B9] Hasenstab-Lehman KE Simpson MG (2012) Cat’s eyes and popcorn flowers: Phylogenetic systematics of the genus *Cryptantha* s.l. (Boraginaceae). Systematic Botany 37(3): 738–757. 10.1600/036364412X648706

[B10] Inventory CNPS (2024) California Native Plant Society, Rare Plant Program, Rare Plant Inventory (online edition, v9.5). https://www.rareplants.cnps.org

[B11] Jepson Flora Project (Eds) (2024) Jepson eFlora. https://ucjeps.berkeley.edu/eflora [accessed 17.05.2024]

[B12] Johnston IM (1925) Studies in the Boraginaceae IV. The North American species of *Cryptantha.* Contributions from the Gray Herbarium of Harvard University 74: 1–114. 10.5962/p.336086

[B13] Johnston IM (1939) Studies in the Boraginaceae, XIII: New or otherwise noteworthy species, chiefly from western United States. Journal of the Arnold Arboretum 20(3): 375–402. 10.5962/bhl.part.21105

[B14] Kahle D Wickham H (2013) ggmap: Spatial Visualization with ggplot2. The R Journal 5(1): 144–161. 10.32614/RJ-2013-014

[B15] Kauffmann M Evens J Kierstead J Murray M Sawyer JO (2022) Chapter 8, Plant communities. In: Kauffmann M Garwood J (Eds) The Klamath Mountains, a natural history.Backcountry Press, Kneeland, CA, 192–239.

[B16] Mabry ME Simpson MG (2018) Evaluating the monophyly and biogeography of *Cryptantha* (Boraginaceae). Systematic Botany 43(1): 53–76. 10.1600/036364418X696978

[B17] Moroni P Simpson MG (2022) Revisión taxonómica del genero *Johnstonella* (Boraginaceae s. str.) en la Argentina. Darwiniana 10(1): 260–270. 10.14522/darwiniana.2022.101.1046

[B18] Moroni P Simpson MG (2023a) Flora Argentina: *Cryptantha* (Boraginaceae). Darwiniana 19: 17–24.

[B19] Moroni P Simpson MG (2023b) Flora Argentina: *Greeneocharis* (Boraginaceae). Darwiniana 19: 36–37.

[B20] Moroni P Simpson MG (2023c) Flora Argentina: *Johnstonella* (Boraginaceae). Darwiniana 19: 56–58.

[B21] Moroni P Martínez A Simpson MG (2021) Nomenclatural revision of *Cryptantha* (Boraginaceae s. str.) names linked to South American taxa. PhytoKeys 181: 29–47. 10.3897/phytokeys.181.69740 34557054 PMC8421323

[B22] Safford H Miller JED (2020) An updated database of serpentine endemism in the California flora. Madrono 27(2): 85–104. 10.3120/0024-9637-67.2.85

[B23] Sawyer JO (2006) Northwest California. University of California Press, Berkeley, California, 1–149. 10.1525/9780520928367

[B24] Simpson MG (2019) Plant Systematics. 3^rd^ edn. Elsevier-Academic Press, New York, 3–16. 10.1016/B978-0-12-812628-8.50001-8

[B25] Simpson MG Kelley RB (2020) *Cryptantha*. In: Meyers SC Jaster T Mitchell KE Harvey T Hardison LK (Eds.) Flora of Oregon.Volume 2: Dicots A–F. Botanical Research Institute of Texas Press, Fort Worth, Texas, U.S.A., 394–403.

[B26] Simpson MG Guilliams CM Hasenstab-Lehman KE Mabry ME Ripma L (2017a) Phylogeny of the popcorn flowers: Use of genome skimming to evaluate monophyly and interrelationships in subtribe Amsinckiinae (Boraginaceae). Taxon 66(6): 1406–1420. 10.12705/666.8

[B27] Simpson MG Johnson LA Villaverde T Guilliams CM (2017b) American amphitropical disjuncts: Perspectives from vascular plant analyses and prospects for future research. American Journal of Botany 104(11): 1600–1650. 10.3732/ajb.1700308

[B28] Simpson MG Hasenstab-Lehman KE Mabry ME Kelley RB (2021) *Cryptantha*. In: Jepson Flora Project (Eds) Jepson eFlora, Revision 9. https://ucjeps.berkeley.edu/eflora

[B29] Stearn WT (1993) Botanical Latin. 4^th^ edn. David & Charles Publishers, Newton Abbot, Devon, Great Britain, 1–546.

[B30] Thiers B (2024) Index Herbariorum. https://sweetgum.nybg.org/science/ih/

[B31] Villagrán C Romo M Castro V (2003) Etnobotánica del sur de los Andes de la Primera Región de Chile: Un enlace entre las culturas altiplánicas y las de quebradas altas del Loa superior. Chungara (Arica) 35(1): 73–124. 10.4067/S0717-73562003000100005

[B32] Weigend M Luebert F Selvi F Brokamp G Hilger HH (2013) Multiple origins for Hound’s tongues (*Cynoglossum* L.) and Navel seeds (*Omphalodes* Mill.) – The phylogeny of the borage family (Boraginaceae s.str.). Molecular Phylogenetics and Evolution 68(3): 604–618. 10.1016/j.ympev.2013.04.009 23608129

